# Interfacial Properties of Monolayer and Bilayer MoS_2_ Contacts with Metals: Beyond the Energy Band Calculations

**DOI:** 10.1038/srep21786

**Published:** 2016-03-01

**Authors:** Hongxia Zhong, Ruge Quhe, Yangyang Wang, Zeyuan Ni, Meng Ye, Zhigang Song, Yuanyuan Pan, Jinbo Yang, Li Yang, Ming Lei, Junjie Shi, Jing Lu

**Affiliations:** 1 State Key Laboratory for Mesoscopic Physics and Department of Physics, Peking University, Beijing 100871, P. R. China; 2 Collaborative Innovation Center of Quantum Matter, Beijing 100871, P. R. China; 3 State Key Laboratory of Information Photonics and Optical Communications & School of Science, Beijing University of Posts and Telecommunications, Beijing 100876, China; 4 Department of Physics, Washington University in St. Louis, St. Louis, Missouri 63130, USA; 5 Department of Nuclear Science and Engineering and Department of Materials Science and Engineering, Massachusetts Institute of Technology, Cambridge, Massachusetts 02139, USA

## Abstract

Although many prototype devices based on two-dimensional (2D) MoS_2_ have been fabricated and wafer scale growth of 2D MoS_2_ has been realized, the fundamental nature of 2D MoS_2_-metal contacts has not been well understood yet. We provide a comprehensive *ab initio* study of the interfacial properties of a series of monolayer (ML) and bilayer (BL) MoS_2_-metal contacts (metal = Sc, Ti, Ag, Pt, Ni, and Au). A comparison between the calculated and observed Schottky barrier heights (SBHs) suggests that many-electron effects are strongly suppressed in channel 2D MoS_2_ due to a charge transfer. The extensively adopted energy band calculation scheme fails to reproduce the observed SBHs in 2D MoS_2_-Sc interface. By contrast, an *ab initio* quantum transport device simulation better reproduces the observed SBH in 2D MoS_2_-Sc interface and highlights the importance of a higher level theoretical approach beyond the energy band calculation in the interface study. BL MoS_2_-metal contacts generally have a reduced SBH than ML MoS_2_-metal contacts due to the interlayer coupling and thus have a higher electron injection efficiency.

Owing to their excellent properties, two-dimensional (2D) molybdenum disulfide MoS_2_ has attracted much recent attention^1^,^2^,^3^,^4^,^5^,^6^. A variety of prototype devices based on 2D MoS_2_ have been fabricated, such as field-effect transistors (FETs)^7^,^8^,^9^, inverters^10^, fully integrated circuits^11^, sensors^12^, photoelectronic devices^13^, phototransistors^14^,^15^, spintronic devices^16^, and valleytronic devices^17^,^18^. Very recently, wafer-scale high performance 2D MoS_2_ FETs have been fabricated in batch mode, paving the way towards atomically thin integrated circuitry^19^. Among 2D MoS_2_, monolayer (ML) and bilayer (BL) MoS_2_ attract the most attention^2^,^3^,^5^,^6^,^7^,^8^,^9^,^10^,^11^,^12^,^13^,^14^,^15^,^16^,^17^,^18^. They show quite interesting differences and make up a pair of complementary materials: (1) ML MoS_2_ has a larger direct band gap, while BL MoS_2_ possesses a smaller indirect band gap due to the strong interlayer coupling. Correspondingly, photoluminescence is dramatically enhanced in ML MoS_2_^6^,^20^. (2) ML MoS_2_ is inversion asymmetric and serves as an ideal valley Hall insulator (VHI)^1^. By contrast, inversion symmetric BL MoS_2_ is not a VHI, but it can be transformed into a VHI with a tunable valley magnetic moment by a vertical electric field, which destroys the inversion symmetry^5^. (3) Zeeman-like spin splitting is nearly intact by a vertical electric field in ML MoS_2_ but it becomes tunable in BL MoS_2_ because top and bottom MoS_2_ feel different electric potentials^16^.

In a real device, semiconducting 2D MoS_2_ needs a contact with metal electrodes, and a Schottky barrier is often formed in semiconductor-metal interface, which impedes the carrier transport. The formation of low-resistance metal contacts is the biggest challenge that masks the intrinsic exceptional electronic properties of 2D MoS_2_, and many efforts have been made to study 2D MoS_2_-metal contact so as to reduce the Schottky barrier height (SBH)^21^,^22^,^23^. The SBH of a 2D MoS_2_-metal contact depends on the work function of metal and the layer number of MoS_2_. Lower work function metal and more MoS_2_ layer number favor a smaller SBH. For example, there is a significant SBH between Ti and ML MoS_2_^24^,^25^; by contrast, Ti forms an Ohmic contact with BL MoS_2_ at room temperature and a Schottky contact with a small SBH of ~0.065 eV at a low temperature^11^,^26^. Although there are several energy band calculations based on single particle density functional theory (DFT) to examine ML MoS_2_-metal interfaces^22^,^23^,^25^,^27^,^28^, a comprehensive energy band calculation for BL MoS_2_-metal interfaces is still lacking at present.

There are two open issues concerning this validity of the DFT energy band approach to treat the SBH of a transistor. Because the SBH at the metal-semiconductor interfaces depends on the difference between the Fermi level (*E*_*f*_) of the metal and the band edge positions of the semiconductor, the band edge positions of the semiconductor must be accurately determined^29^,^30^. It is well known that the common LDA and GGA fail to do so. From a theoretical point of view, the accurate band edge positions should be the quasiparticle energy, which can be obtained from first-principles many-electron Green function approach within the *GW* approximation, where electron-electron correlation effects are treated properly^13^,^31^,^32^,^33^,^34^,^35^,^36^,^37^,^38^. The first open issue concerning the DFT energy band scheme to evaluate SBH is whether the many-electron effects should be included.

The second open issue is the way of the energy band calculation in treating the SBH of a transistor. There are two possible interfaces to form Schottky barrier in a MoS_2_ transistor^23^,^39^: one is the source/drain interface (B) between the contacted MoS_2_ and the metal surface in the vertical direction if the interaction between MoS_2_ and metal is weak, and the other is source/drain-channel (D) interface between the contacted MoS_2_ and channel MoS_2_ in the lateral direction if a metallization has taken place between MoS_2_ and metal. The energy band calculation treats the source and the channel independently and ignores the coupling between the source and the channel, which may lead to the Fermi level pinning and change the SBH.

In this Article, we provide a theoretical study of the interfacial properties of ML and BL MoS_2_ on several commonly used metals (Sc, Ti, Ag, Pt, Ni, and Au)^8^,^21^ at different levels. A comparison between the observed and calculated SBH in ML and BL MoS_2_-Ti interfaces suggests that *GW* correction to the band edge positions of 2D MoS_2_ is strongly depressed in a device because of a charge transfer. More importantly, we find that the energy band calculation is unable to reproduce the observed SBHs in 2D MoS_2_-Sc and -Pt interfaces. This failure prompts us to perform direct an *ab initio* quantum transport device simulation, and we find the SBHs in 2D MoS_2_-Sc and -Pt interfaces can be better reproduced in latter calculation. SBH is found to be reduced from ML MoS_2_-metal interfaces to BL MoS_2_-metal interfaces in different level calculations.

## Methodology

We use six layers of metal atoms (Ni, Ag, Pt, and Au in (111) orientation and Sc and Ti in (0001) orientation) to model the metal surface and construct a supercell with ML and BL MoS_2_ adsorbed on one side of the metal surface. BL MoS_2_ takes AB stacking mode (with a *D*_3*d*_ point group symmetry) in our model. The calculated in-plane lattice constant *a* = 3.166 Å, which is in good agreement with the experimental value 3.160 Å^40^. The MoS_2_ 1 × 1 unit cell is adjusted to the 1 × 1 unit cells of Sc and Ti(0001) faces, and the MoS_2_ 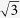 × 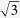 unit cell is adjusted to 2 × 2 unit cells 27. The lattice constant mismatches with respect to that of MoS_2_ are 1.2~9.1%. A vacuum buffer space of at least 15 Ǻ is set to ensure decoupling between neighboring slabs. MoS_2_ mainly interacts with the topmost three layers metal atoms^22^, so cell shape and the bottom three layers of metal atoms are fixed.

The geometry optimization and electronic properties of the periodic structures are performed using the projector augmented wave (PAW) method implemented in the Vienna *ab initio* simulation package (VASP) code^41^,^42^. The generalized gradient approximation (GGA) functional to the exchange-correction functional, of the Perdew–Wang 91 (PW91) form^43^ with vdW corrections (VDW-DFT)^44^, and the PAW pseudopotential are adopted^42^. The cut off energy is set to 500 eV after convergence tests. An equivalent Monkhorst-Pack *k*-points grid^45^ of 25 × 25 × 1 for a MoS_2_ unit cell is chosen for supercell relaxations and 30 × 30 × 1 for property calculations. In our current calculations, the total energy is converged to less than 10^−5^ eV. Dipole corrections in the *z* direction are used in all calculations. The maximum force is less than 0.02 eV/Å during optimization. We employ the GW band gap calculated by Louie’s group^36^ and experimental band gap center (BGC)^46^ to analyze the SBH, because the values do not change much in different groups^37^,^47^,^48^.

Two-probe model is established to study the interface properties in a FET configuration. The devices are constructed of ~60 Å ML/BL MoS_2_ in the channel region along the transport direction and ML/BL MoS_2_-Sc (Pt) interfaces in the electrode region. The electrode is consisted of 6 Sc (Pt) layers with ML/BL MoS_2_ adsorbed on the Sc (0001) (Pt(111)) surface and a ~15 Ǻ vacuum buffer space. The supercell of the electrode region contains 1 × 1 unit cell of MoS_2_ and Sc (0001) surface in for Sc electrode and 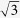 × 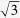 unit cell of MoS_2_ and 2 × 2 unit cells of Pt(111) surface for Pt electrode. The transport properties of the FET are calculated by the DFT coupled with the nonequilibrium Green’s function (NEGF) method, as implemented in the ATK 11.8 package^49^,^50^. We employ the single-zeta plus polarization (SZP) basis set during the device simulation. A test using higher double-zeta plus polarization (DZP) basis set is also performed. In consistent with previous DFT calculations, GGA of PW91 form to the exchange-correlation functional is used through the device simulations. The Monkhorst-Pack *k*-point meshes for the central region and electrodes are sampled with 1 × 50 × 1 and 50 × 50 × 1 separately. The temperature is set to 300 K. The Neumann condition is used on the boundaries of the direction vertical to the MoS_2_ plane. On the surfaces connecting the electrodes and the central region, we employ Dirichlet boundary condition to ensure the charge neutrality in the source and the drain region. The transmission coefficient T(*E*) is given by T(*E*) = *G*(*E*)Γ^L^(*E*)*G*^†^(*E*)Γ^R^(*E*), where *G*(*E*) and *G*^†^(*E*) are the retarded and advanced Green functions, and the broadening function Γ^L/R^(*E*) describes the level broadening due to left/right electrode and is obtained from the electrode self-energies Γ_L/R_(*E*) = *i*(Σ_L/R_ −Σ^†^_L/R_)). The electrode self-energies can be viewed as an effective Hamiltonian describing the interaction between device and lead.

## Results and Discussion

### Geometry and stability of ML and BL MoS_2_-metal interfaces

**Figure 1:**
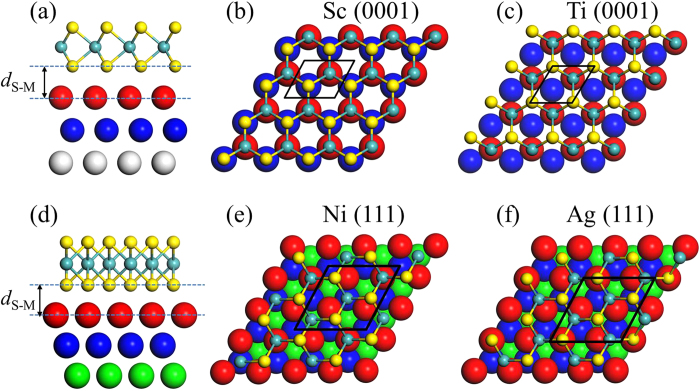
Interfacial structures of the most stable configuration for ML MoS_2_ on metal surfaces. (**a**) Side and (**b**) top views of ML MoS_2_ on Sc(0001) surface. (**c**) Top view of MoS_2_ on Ti(0001) surface. (**d**) Side and (**e**) top views of ML MoS_2_ on Ni and Pt(111) surfaces. (**f**) Top view of MoS_2_ on Ag and Au(111) surfaces. *d*_S-M_ is the equilibrium distance between the metal surface and the bottom layer MoS_2_. The rhombi plotted in black line shows the unit cell for each structure.

After optimizing the structures from 6 initial configurations in an interface with 1 ×1 MoS_2_ unit cell and 3 initial configurations in an interface with 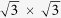 MoS_2_ unit cell, we obtain the most stable configurations of the ML MoS_2_-metal interfaces, as shown in Fig. 1. The initial configurations of BL MoS_2_-metal interfaces are constructed on the basis of the most stable ML MoS_2_-metal interfaces. On Sc(0001), the Mo atoms in the primitive cell sit above the top metal atom layer, and the S atoms sit above the second MoS_2_ metal atom layer metal atom; on Ti(0001), the Mo atoms in the primitive cell still sit above the top metal atom layer, but the S atoms sit above the centers of triangles. On Ni and Pt(111), the three Mo atoms in the supercell sit above the fcc hollow, hcp hollow, and top sites, respectively. In the cases of Ag and Au(111), the Mo atoms are all above the centers of the triangles formed by the fcc, hcp, and top sites. The calculated key parameters of ML and BL MoS_2_-metal interfaces studied in this work are summarized in Table 1. When ML and BL MoS_2_ are in contact with metals, the equilibrium distances *d*_S-M_ range from 1.557~3.405 Å, increasing in the order of Ti < Sc < Ni < Pt < Ag < Au. The binding energies *E*_b_ have a reversal order, i.e., Ti > Sc > Ni > Pt > Ag > Au, since a smaller *d*_S-M_ generally causes a larger binding energy. Ti and Sc have a strong adhesion with ML/BL MoS_2_ (*E*_b_ = 1.181~1.848 eV per surface sulfur atom), Ni, Pt, and Ag have a medium adhesion (*E*_b_ = 0.503~0.830 eV per surface sulfur atom), and Au has a weak adhesion (*E*_b_ = 0.307~0.354 eV per surface sulfur atom). ML and BL MoS_2_-metal contacts nearly share the same *d*_S-M_ and *E*_b_. The binding of MoS_2_ to metal surfaces^22^,^27^ is considerably stronger than that of graphene to metal surfaces, with the binding energy of 0.027~0.327 eV per carbon atom^51^. Such a difference is reasonable because MoS_2_ is chemically more reactive than graphene. We note that previous DFT calculations indicate that the *E*_b_ of ML MoS_2_ on Ir, Pd, and Ru surfaces ranges from 0.62~0.82 eV per surface sulfur atom^27^.

### Electronic structure of ML and BL MoS_2_-metal interfaces

**Figure 2:**
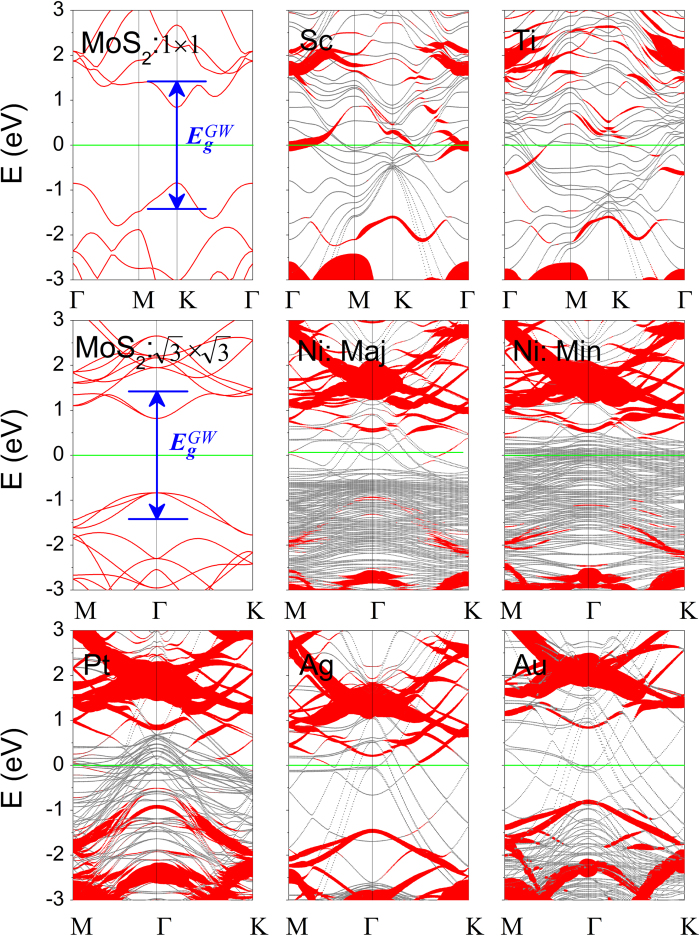
Band structures of ML MoS_2_ on Sc, Ti, Ni, Pt, Ag, and Au surfaces by the DFT method, respectively. The Fermi level is at zero energy. Gray line: metal surface bands; red line: bands of MoS_2_. The line width is proportional to the weight. Blue line: the positions of CBM and VBM of MoS_2_ after the *GW*-BGC correction. The labels Maj/Min indicate the majority-spin and minority-spin bands of MoS_2_ on Ni surface. The band structure of free-standing ML MoS_2_ calculated in a primitive unit cell and a 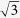 × 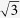 supercell are provided for comparison.

**Figure 3:**
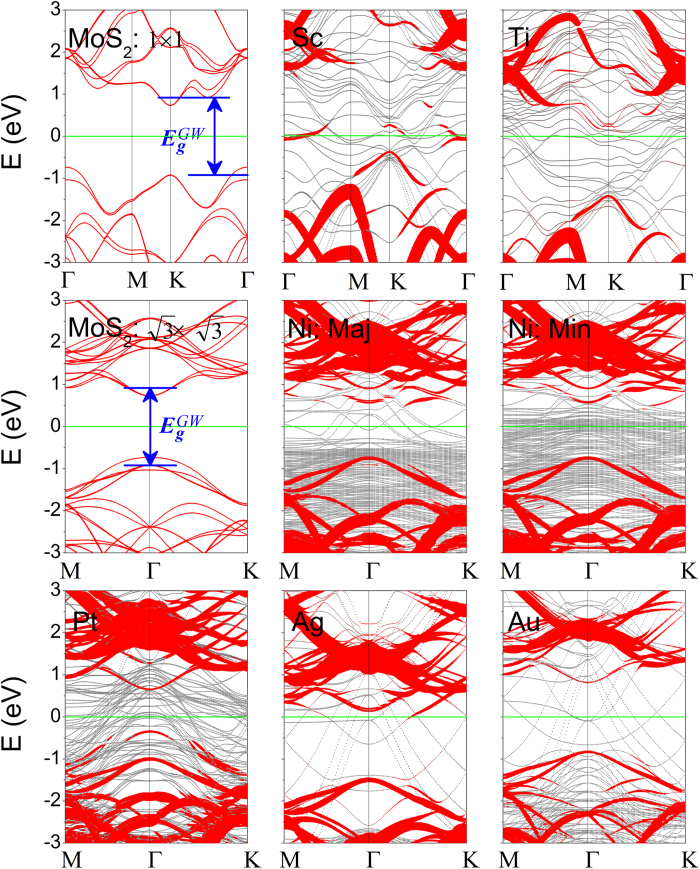
Band structures of BL MoS_2_ on Sc, Ti, Ni, Pt, Ag, and Au surfaces by the DFT method, respectively. The Fermi level is at zero energy. The gray (red) line denotes metal surface (BL MoS_2_) bands. The line width is proportional to the weight. Blue line: the positions of CBM and VBM of MoS_2_ at the *GW*-BGC scheme. The labels Maj/Min indicate the majority-spin and minority-spin bands of BL MoS_2_ on Ni surface. The band structure of free-standing BL MoS_2_ calculated in a primitive unit cell and a 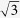 × 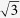 supercell are provided for comparison.

The electronic structures of free-standing ML MoS_2_ and the interfacial systems are presented in Fig. 2. Free-standing ML MoS_2_ has a direct band gap of 1.68 eV, consistent with the reported PBE value of 1.67 eV^52^. The band structures of ML MoS_2_-metal contacts are classified into three categories in terms of the hybridization degree of ML MoS_2_ on metals. The band structure of ML MoS_2_ is identifiable clearly for MoS_2_ on Au surface (weak hybridization), as a result of weak charge-transfer interaction and dispersion interaction between ML MoS_2_ and Au surface. The band structure of ML MoS_2_ is destroyed seriously (strong hybridization) by Sc and Ti surfaces and is destroyed but still identifiable (medium hybridization) by Ni, Pt, and Ag surfaces, because the outmost electrons of the five metals except Ag are *d* electrons, which strongly hybridize with the states near the Fermi level *E*_*f*_ of ML MoS_2_. For the sake of comparison, the electronic structures of free-standing BL MoS_2_ and BL MoS_2_-metal interfaces are also shown in Fig. 3, with a smaller indirect band gap of 1.46 eV for free-standing BL MoS_2_. The band hybridization degree is similar from ML to BL MoS_2_ and can be also divisible into the same three categories. The hybridization degree of ML/BL MoS_2_ on metals is consistent with its binding energy: The higher the binding energy is, the higher the hybridization degree is.

**Figure 4:**
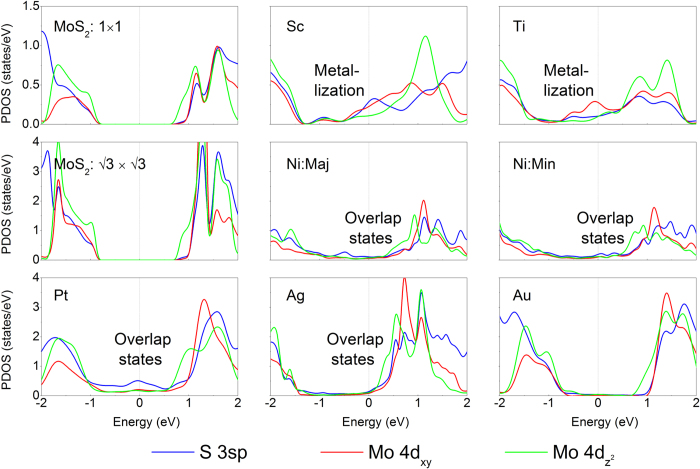
Partial density of states (PDOS) (DOS on specified atoms and orbitals, for example, Mo-*d* (*d*-orbital on Mo)) of ML MoS_2_ on Sc, Ti, Ni, Pt, Ag, and Au surfaces at the DFT level. The Fermi level is at zero energy. The PDOS of free-standing ML MoS_2_ calculated in a primitive unit cell and a 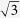
×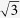 supercell is provided for comparison.

**Figure 5:**
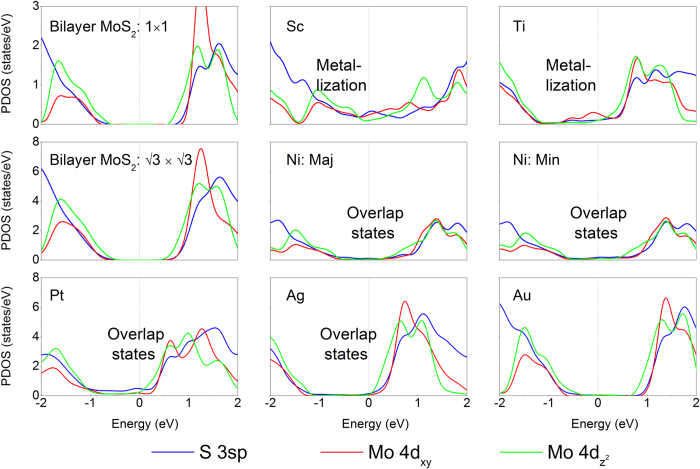
PDOS of BL MoS_2_ on Sc, Ti, Ni, Pt, Ag, and Au surfaces at the DFT level. The Fermi level is at zero energy. The PDOS of free-standing BL MoS_2_ calculated in a primitive unit cell and a 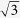 × 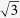 supercell are provided for comparison.

In order to have a deep understanding of the hybridization in Figs 2 and 3, we further calculate the partial density of states (PDOS) on Mo and S orbitals for ML and BL MoS_2_-metal contacts as shown in Figs 4 and 5. Upon making a contact with Sc and Ti, a large amount of Mo and S states are extended into the original band gap of ML/BL MoS_2_ due to metallization. In the MoS_2_-Sc system, the contribution of S 3*sp* and Mo 4*d* states dominate *E*_*f*_, which is associated with a strong S-Sc mixing. *E*_*f*_ is dominated by Mo 4*d* states, with the other states playing a minor role in the MoS_2_-Ti system. Mo and S states also appear in the original MoS_2_ band gap due to orbital overlap between MoS_2_ and metal. There is no Mo and S state in the original MoS_2_ band gap in MoS_2_-Au system, indicating that MoS_2_ preserves the semiconducting nature on Au surface.

**Figure 6:**
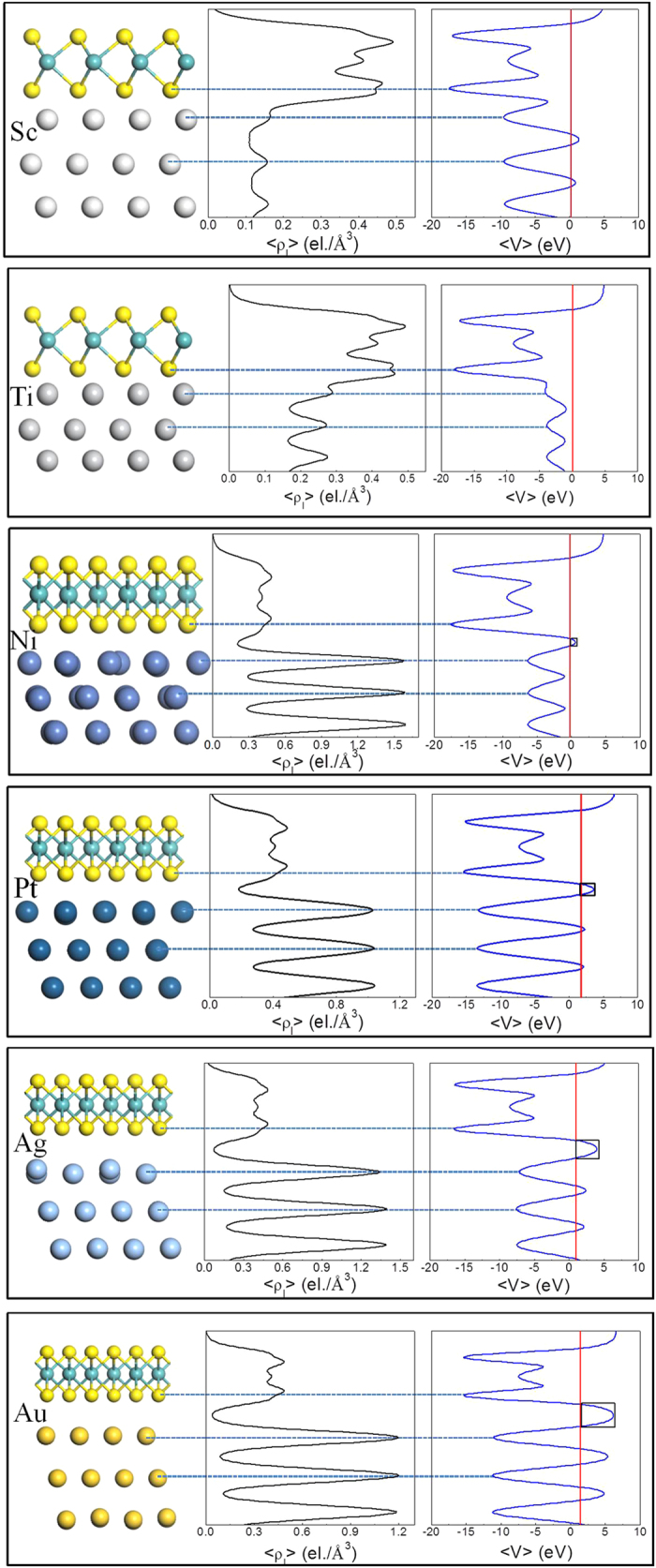
Electronic structure at the interface between ML MoS_2_ and metal at the DFT level. <*ρ*_*l*_> is the average value in planes parallel to the interface of MoS_2_-metal. 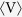 is the average electrostatic potential in planes normal to the MoS_2_-metal interface. The dot lines indicate the location of the sulfur layer and the metal layers at the interface. The higher the *ρ*_*l*_at the interface is, the higher the electron injection is.

Large charge carrier density at the source/drain interface B indicates a strong overlap of electron orbitals and sufficient injection of electron into the MoS_2_ layer^22^. The electron densities averaged in planes parallel to the interface *ρ*_l_ of the investigated six ML MoS_2_-metal contacts are displayed in Fig. 6. We can see from Fig. 6 that *ρ*_l_ at the strong bonding interfaces (Sc, Ti, Ni, and Pt) is higher than that at the weak bonding interfaces (Ag and Au), a difference compared with the PDOS analysis in Fig. 4. This difference implies that the chemisorption interface has a larger possibility to achieve a lower contact resistance.

### Many-electron effects

The accurate SBH at a metal-semiconductor interface depends on the absolute band-edge positions of the semiconductor. Because the DFT method seriously underestimates the band gap of a semiconductor, the inclusion of the *GW* correction is also necessary to obtain a correct band gap and absolute band-edge positions of a freestanding (or undoped) semiconductor. If the band gap center (BGC) or Fermi level *E*_*f*_ or work function and the *GW* corrected band gap (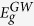) of the semiconductor are available, the absolute energies at the conduction band maximum (CBM) and the valence band minimum (VBM) can be obtained via the relation:


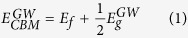



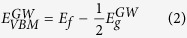


**Figure 7:**
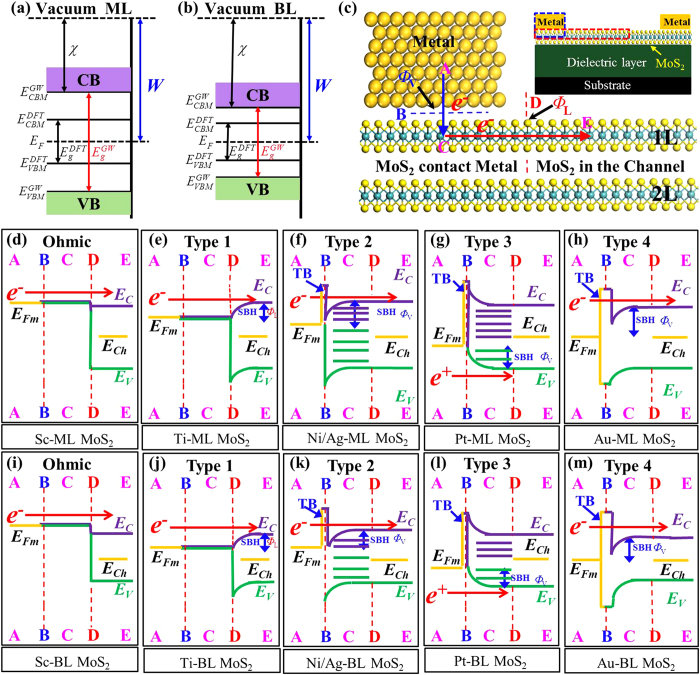
Schematic illustration of the absolute band positions with respect to the vacuum level at both DFT and *GW* levels for ML (**a**) and BL (**b**) MoS_2_, respectively. (**c**) Schematic cross-sectional view of a typical metal contact to 2D MoS_2_. A, C, and E denote the three regions while B and D are the two interfaces separating them. Blue and red arrows show the pathway (A → B → C → D→ E) of electron injection from contact metal (A) to the MoS_2_ channel (E). Inset figure shows the typical topology of a MoS_2_ FET. (**d**–**m**) Ten band diagrams of (**c**) at the DFT level, depending on the type of metals and MoS_2_ layer number. TB denotes the tunneling transmission barrier. Examples are provided at the bottom (top) of each diagram. *E*_*Fm*_ and *E*_*Ch*_ denote the Fermi level of the absorbed system and the band gap center of channel MoS_2_, respectively. Red arrows indicate the direction of electron or hole flow. The cause of the band bending is given in the main text.

Unfortunately, in many cases, the BGC of a semiconductor is unavailable. A common solution^29^,^30^ is to assume that the BGC at the DFT level is unchanged after the *GW* correction (named *GW*-BGC approximation). Figure 7(a,b) illustrate the *GW* correction to the absolute band positions for freestanding ML and BL MoS_2_ in BGC approximation. Based on the *GW*-BGC scheme, the calculated ionization potential (IP = 5.45 eV) and electron affinity (χ = 4.22 eV) of bulk MoS_2_, compared with values of 5.33 and 4.45 eV at the DFT level^29^, are in good agreement with the experimental values (IP = 5.47 ± 0.15 eV and χ = 4.07 ± 0.35 eV)^53^. Actually, Yang *et al.*^54^ found that the absolute band-edge energies for ML dichalcogenides given by the direct *GW* method and the *GW*-BGC scheme are quite similar. Therefore, the *GW-*BGC approximation is a good approximation for our studied MoS_2_ systems.

The *GW* corrections to the band gap of free-standing ML (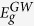 = 2.84 eV) and BL (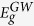 = 1.82 eV) MoS_2_ are available^36^,^37^. In our calculations for the SBH at the vertical direction, we take the *GW*-BGC approximation to determine the absolute band edge positions. In our calculations for the SBH at the lateral direction, we determine the *GW-*corrected absolute band position by taking the experimental work function (5.25 eV^46^) of free-standing BL MoS_2_ (namely channel BL MoS_2_) as the BGC and further assume free-standing ML and BL MoS_2_ share identical BGC. The calculated work function of free-standing BL MoS_2_ at the DFT level is 5.04 eV, which is 0.21 eV slightly smaller than its experimental value. The calculated work function of free-standing ML MoS_2_ at the DFT level is merely 0.08 eV larger than the calculated one of free-standing BL MoS_2_.

### Schottky barrier at ML and BL MoS_2_-metal interfaces

The vertical *n*-type (or *p*-type) Schottky barrier for the medium (Ag, Pt, and Ni) and weak (Au) bonding cases can be obtained from the difference between *E*_*f*_ and the identifiable CBM (or VBM) of ML/BL MoS_2_ of the interfacial system, which are shown in the same band structures. By contrast, a strong band hybridization has taken place for ML/BL MoS_2_ on Sc and Ti surfaces, resulting in metallization of ML/BL MoS_2_ and absence of vertical Schottky barrier for the four contacts. It has been proved that for the semiconductor fully under metal (namely, in the electrode region), the many-electron effects are greatly depressed if a charge transfer takes place^38^,^55^; as a result, the KS band edge positions and band gap are a good approximation. Therefore, we only consider many-electron effects for the band structure of the semiconductor in the channel of a device in the case that a metallization takes place between metal and underneath 2D MoS_2_. Namely, only as calculating the lateral SBH of 2D MoS_2_-Sc and -Ti contacts, we consider many-electron effects. We obtain electron SBH at the DFT level of 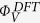 = 0.212 and 0.633 eV for ML MoS_2_-Ag and Ni contacts, respectively, from the difference between *E*_*f*_ and the identifiable CBM of ML MoS_2_ shown in the same band structure (Fig. 2). Similarly, we obtain hole SBH of 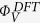 = 0.520 eV for ML MoS_2_-Pt contact from the difference between *E*_*f*_ and the identifiable VBM of ML MoS_2_. While for ML MoS_2_-Au contact, *E*_*f*_ is nearly in the middle of the band gap; Therefore, ML MoS_2_-Au contact has a midgap Schottky barrier, and this is consistent with the experiment^56^.

Lateral Schottky barrier *Φ*_L_ is determined by the energy difference between *E*_*f*_ of the interfacial system and the CBM (*n*-type) or VBM (*p*-type) of channel ML MoS_2_. ML MoS_2_ forms an Ohmic contact with Sc in the lateral direction at the DFT level since *E*_*f*_ of the interfacial system is higher than the 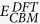 of channel MoS_2_. However, there is a large lateral SBH at the *GW* level, with 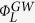 = 0.539 eV. There is a lateral *n*-type Schottky barrier for Ti contacts at both the DFT and *GW* levels, with smaller 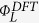 = 0.216 and larger 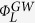 = 0.796 eV. The DFT SBHs of ML MoS_2_-metal interfaces are in good agreement with the previous DFT calculations (see Table 1). For example, the lateral DFT SBH for ML MoS_2_-Ti interface is 0.33 eV calculated by Banerjee *et al.*^23^,^39^. There is some uncertainty in identifying the metallization. However, even if we identify a metallization for ML MoS_2_ under Ag, Pt, and Ni, the values of the resulting lateral SBHs are close to those of the vertical SBHs.

The vertical Ohmic contact feature remains on Sc and Ti surfaces from ML to BL MoS_2_, because the strong band hybridization remains. From ML to BL MoS_2_, *Φ*_V_ in MoS_2_-Au contact is significantly decreased by 0.096 eV at the DFT level as a result of the reduction of the band gap (0.220 eV). The vertical SBHs for Ag, Pt, and Ni contacts are slightly decreased by 0.074, 0.175, and 0.021 eV, respectively, at the DFT level from ML to BL MoS_2_. The reduced SBH from ML to BL MoS_2_ is in good agreement with the experiment^21^. BL MoS_2_ still forms an Ohmic contact with Sc in the lateral direction.

Since the lattice mismatches are large for the Sc-MoS_2_ (4.485%) and Ti-MoS_2_ (6.791%) interfaces in the above study, we further enlarge the supercell to reduce the lattice mismatch. The 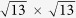 unit cell of MoS_2_ is adjusted to the 2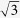 × 2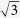 unit cells of Sc(0001) surface, with the lattice mismatch decreased to 1.29%. The 2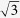 ×2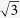 unit cell of MoS_2_ is adjusted to 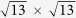 unit cells of Ti(0001) surface, with the lattice mismatch decreased to 2.99%. Compared with the large mismatch configuration, the small mismatch ones do not change the contact type and just slightly increase 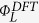
from 0.187 (0.096) to 0.216 (0.161) eV for ML (BL) MoS_2_-Ti contact, which is closer to a DFT value of 0.33 eV of Banerjee *et al.*^23^,^39^ based on a larger ML MoS_2_-Ti interfacial supercell containing 6 Mo and 12 S atoms per unit cell in the contact region.

The experimentally extracted SBHs of ML and BL MoS_2_-Ti contact are 0.3~0.35^23^ and 0.065 eV^26^, respectively, which are in agreement with our calculated values of 0.216 and 0.161 eV at the DFT level but apparently deviate from the corresponding values with many-body effect correction (0.796 and 0.341 eV, respectively). Such a comparison suggests that many-electron effects have been greatly depressed by the charge transfer between channel MoS_2_ and the electrodes, which significantly screens the electron-electron Coulomb interaction and validates sing-electron approximation. In other word, the transport gap of ML MoS_2_ is determined by the DFT band gap rather than the quasiparticle band gap.

In a recent work, the SBH and the transport gap of phosphorene have been measured^57^. Phosphorene is *p*-type doped by Ni electrodes, and the transport gaps of ML and few layer phosphorene with Ni electrodes are in good agreement with the DFT band gaps at the GGA level. For example, the transport gap of ML and BL phosphorene are 0.98 ± 0.4 and 0.71 ± 0.4 eV^57^, respectively, and the corresponding band gaps are 0.91 and 0.6 eV at the DFT level^58^, while the quasiparticle band gaps are 2.0 and 1.3 eV^59^. Therefore, the suppressed many-electron effects can be expanded to a general device if 2D channel semiconductor is doped by electrodes, and correspondingly the transport gap depends on the DFT band gap instead of the quasiparticle band gap.

In our above calculations, we adapt the lattice constant of MoS_2_ to that of metal surfaces as the match way in ref. 27 in view of the fact that the bulk metal electrode is more robust than ML and BL MoS_2_. We note that the lattice constant of MoS_2_ is fixed in ref. 28. In order to explore the effects of the match way on the work function of MoS_2_-metal interface, we give the work function of interfacial systems in the case of fixing MoS_2_ lattices in Table S1. The work function of the system with Ti surface adjusted to MoS_2_ is 0.205 eV smaller than that of the system with BL MoS_2_ adjusted to Ti surface; consequently, the lateral SBH disappears. Such a result is in consistent with the experimental SBH of 0.065 eV for BL MoS_2_-Ti contact^26^. There is nearly no difference in work function between these two strained method for BL MoS_2_-Sc and ML MoS_2_-Ti contacts.

### Tunneling barrier at ML and BL MoS_2_-metal interfaces

In order to complete the analysis of contacts, we further investigate the electrostatic potential at the ML MoS_2_-metal interfaces and show the results in Fig. 6. The tunneling barrier ∆*V* here is defined as the potential energy above the Fermi energy between the MoS_2_ and metal surfaces, indicated by the black rectangular, and the tunneling width *w*_B_ is defined as the full width at half maximum of the ∆*V*. As shown in Fig. 6 and Table 2, the ∆*V* values at the strong hybridization interfaces (Sc, Ti, Ni, and Pt) are significantly lower and the *w*_B_ values are significantly narrower than those at the weak ones (Ag and Au). A lower barrier height and a narrower width at a semiconductor-metal interface mean a higher electron injection efficiency. We estimate the tunneling probabilities *T*_B_ from metal to MoS_2_ using a square potential barrier model as:


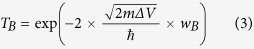


where *m* is the effective mass of a free electron and *ħ* is the Plank’s constant. The *T*_B_ values are thus estimated to be 100, 100, 74.33, 53.21, 19.68, and 4.74% for Sc, Ti, Ni, Pt, Ag, and Au contacts, respectively (see Table 2). Apparently, Sc and Ti contacts have perfect transmission. The tunneling properties of the tunneling barrier at the BL MoS_2_-metal interfaces are also summarized in Table 2. Compared with the case of ML MoS_2_ contact metals, there is little change in the tunneling properties for BL MoS_2_, indicating that the tunneling properties are insensitive to the MoS_2_ layer number.

In the light of Schottky barrier and tunneling barrier, the nature of MoS_2_- metal contacts can be classified into five types. Sc can form high quality contact interface with ML and BL MoS_2_ with zero tunneling barrier and zero Schottky barrier, leading to Ohmic contact (Fig. 7(d)). Although the metallization of ML MoS_2_ with Ti eliminates the Schottky barrier at the interface B, the injected electrons from the metal still confront a *n*-type Schottky barrier at the interface D, leading to Type 1 in Fig. 7(e). The nature of BL MoS_2_-Ti contacts also belong to Type 1. It is noteworthy that the tunneling barrier vanishes in Type 1 contact due to the metallization at interface B. Unlike the case in Type 1, there is a tunneling barrier at the interface B in Types 2 and 3 contacts. Only *p*-type Schottky barrier is formed in ML and BL MoS_2_-Pt contacts (Type 3, Fig. 7(g)). In Type 4 contact (ML and BL MoS_2_-Au), Schottky barrier and tunneling barrier are formed at the interface B, and SBH is zero at the interface D because of the lack of orbital overlaps.

### Fermi level line-up

**Figure 8:**
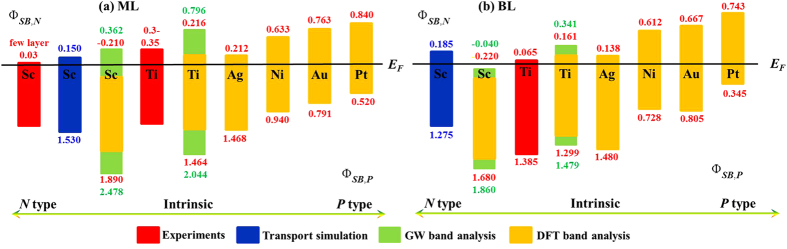
SBHs of (**a**) ML and (**b**) BL MoS_2_ on the six metal surfaces. 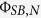 denotes *n* type SB for electrons, while 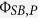 represents *p* type SB for holes.

Our calculated 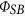 of ML MoS_2_ on all investigated metal surfaces are listed in Fig. 8(a), in which the *GW* results for Sc and Ti contacts are also provided for comparison. For Sc contacts, the 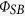 obtained by transport calculations is also presented, which will be discussed later in the transport properties. The CBM of Sc, Ti, Ag, Au, and Ni-ML MoS_2_ systems are closer to the Fermi levels than the VBM, leading to the *n*-type contact. While the VBM of Pt-ML MoS_2_ absorbed system is closer to the Fermi levels and form *p*-type contact. The *n*-type characteristic of ML MoS_2_ on Sc, Ti, Ag, Au, and Ni surfaces has been observed experimentally^8^,^21^,^24^, and the *p*-type characteristic on Pt surface is calculated in other DFT calculation^28^. Therefore, ML MoS_2_ *p−n* junction can be fabricated by using Sc, Ti, Ag, Au, or Ni to contact one end of ML MoS_2_ and Pt to contact the other end of it. The ML MoS_2_ *p−n* junction can be used to develop optoelectronics or valley-optoelectronics technology^60^. Comparing the 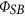 at the DFT and *GW* levels for Sc and Ti contacts, we find that the doping type is unchanged.

The calculated 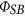 of BL MoS_2_ on the six metal surfaces are listed in Fig. 8(b), in which the *GW* results for Sc and Ti contacts are also provided for comparison. Compared with Fig. 8(a), the *GW* correction to the band gap of BL MoS_2_ is less significant because many-body effect is reduced with the increasing size in the vertical direction. BL MoS_2_ FET is also *p*-type doped by Pt contact and *n*-type doped by the other five contacts. Consistently, the experiments have found *n*-type characteristic of BL MoS_2_ on Ti and Au surfaces^11^,^26^.

**Figure 9:**
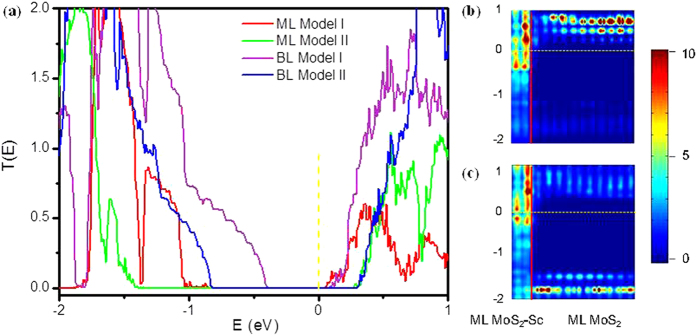
(**a**) Transmission spectra of the ML and BL MoS_2_ transistors with Sc electrodes. In Model I, the lattice constant of MoS_2_ is adjusted to that of Sc, while in Model II the lattice constant of Sc is adjusted to that of MoS_2_. (**b,c**) Local density of states (LDOS) in color coding for the ML MoS_2_ transistors in Models I and II, respectively. The red line indicates the boundary of ML MoS_2_-Sc and the free-standing ML MoS_2_, and the yellow dashed line indicates the Fermi level.

The Fermi level shift Δ*E*_*f*_ is defined as the difference between the interfacial systems and free-standing 2D MoS_2_ work functions when the band hybridization occurs at the interfaces (Sc, Ti, Ni, Pt, and Ag contacts). Δ*E*_*f*_ is defined as Δ*E*_*f*_ = *E*_mid_ – *E*_*f*_ for the interface without band hybridization (for Au contact), where *E*_mid_ is the midpoint of the identifiable band gap of MoS_2_. Negative (positive) Δ*E*_*f*_ means *n*-type (*p*-type) doping of 2D MoS_2_. The doping types determined from Δ*E*_*f*_ are in agreement with those determined from Figs 8 and 9. Δ*E*_*f*_ as a function of the difference between the clean metal (*W*_M_) and ML (BL) MoS_2_ work functions (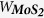) is shown in Fig. S1 (2). The cross point from *n*- to *p*-type doping is *W*_M_ – 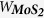 = 0.21 (0.13) eV for ML (BL) case. The Δ*E*_*f*_ has a nearly linear dependence with the *W*_M_ –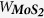
with a slope of 0.64 in both ML and BL MoS_2_-metal contacts, approximately equal to the previously reported theoretical value of 0.71 in ML MoS_2_-metal contacts^28^. Notably, Δ*E*_*f*_ is insensitive to the MoS_2_ layer number, leading to the same linear relation between Δ*E*_*f*_ and work function. Note that the slope close to 0 indicates a strong Fermi level pinning, and we therefore observe a partial Fermi level pinning picture once more when the six metals contact ML MoS_2_. The partial Fermi level pinning is a synergic result of the metal work function modification and the interface gap states formation in the studied interface systems^28^.

### Quantum Transport Simulation

We note that the experiment reported that few layer (3–18 layers) MoS_2_-Sc contact still has a very small SBH of 0.03 eV^21^. ML and BL MoS_2_ should have a larger SBH due to the enhanced band gaps compared with few layer MoS_2_ and this is inconsistent with the predicted Ohmic contact for Sc electrode (*E*_*f*_ of Sc electrode is above the CBM of channel ML/BL MoS_2_ by 0.22/0.21 eV) in the above dual interface model calculation. In the dual interface model, one determines the SBH indirectly by calculating the work functions of MoS_2_ under metal and channel MoS_2_ separately. In a real device, there is possible complex coupling between MoS_2_ under metal and channel MoS_2_ (namely Fermi level pinning). A direct and better theoretical approach to determine the SBH of a FET is to calculate the transport property of the device by using the DFT method coupled with NEGF.

In our quantum transport simulations, the device is constructed of ML/BL MoS_2_ in the channel region and ML/BL MoS_2_-Sc interfaces in the electrode region. The lattice constant of the ML/BL MoS_2_ should be carefully chosen, as it directly affects the size of the band gap and thus transport properties. In a real device, the lattice constant of the ML/BL MoS_2_ in the central region is close to that of free-standing ML/BL MoS_2_, while in the electrode region the lattice constant of the ML/BL MoS_2_ should be adapted to that of corresponding bulk metals supercell. In order to capture this feature, we consider two extreme cases in the transport calculations: in Model I, the lattice constant of ML/BL MoS_2_ in the device is adapted to that of Sc surface, and in Model II, the lattice constant of Sc surface is adapted to that of ML/BL MoS_2_. One could expect that the transport properties of the real device should be between the two cases.

The transmission spectra of ML and BL MoS_2_ transistors using the two models calculated with SZP basis set are provided in Fig. 9(a), respectively. A test shows that a larger DZP basis set gives a quite close SBH. The transmission spectra of ML MoS_2_ transistors give transport gaps of 0.92 eV in Model I and 1.67 eV in Model II, and the latter value is quite close to the band gap (1.68 eV) of free-standing ML MoS_2_. The Fermi level *E*_*f*_ is slightly below the CBM in both two models, showing a *n*-type Schottky barrier between ML MoS_2_ and Sc electrode in the devices. The values of the electron SBH are read as 0.040 eV and 0.260 eV in Models I and II, respectively. As the real system is between the two extreme cases, we estimate the SBH in the real ML MoS_2_ transistor with Sc electrodes to be around 0.150 eV by roughly averaging the values of the two cases. As the number of MoS_2_ layers increases, its band gap decreases. Our transport simulations also show a reduction (~0.09 eV in Model I and 0.56 eV in Model II, respectively) of the transport gap of BL MoS_2_ compared to that of ML. The average value of SBH in the BL MoS_2_ with Sc contact over the two models is estimated to 0.185 eV in the transport simulation. Therefore, increasing the lay number of MoS_2_ not always leads to a decrease in SBH although it often does.

The local density of states (LDOS) versus the coordinate along the transport direction of ML MoS_2_ transistors using the two models calculated with SZP basis set are provided in Fig. 9(b,c). It is apparent from the LDOS that the conduction band is bent downward due to an electron transfer from Sc to channel ML MoS_2_ where no impurity states exist. Such a downward bending is different from a common band upward bending in a metal-*n* type semiconductor interface where donor states exist and electrons are transferred from semiconductor to metal. In accordance with the value calculated from the transmission spectra, the LDOS also shows an average *n*-type SBH of 0.15 eV for ML MoS_2_-Sc interface. Taken together, unlike the DFT energy band analysis, which gives an artificial Ohmic contact, the quantum transport simulations give a *n*-type Schottky contact for ML and BL MoS_2_ Sc-interfaces with electron SBH of 0.150 and 0.185 eV, respectively, which are qualitatively in agreement with the experiment^21^, where 3–18 layer MoS_2_ Sc-interface has a small electron SBH of 0.03 eV.

The failure of the energy band analysis in predicting MoS_2_-Sc contact comes from the ignorance of the coupling between MoS_2_ under Sc and channel MoS_2_ because we calculate the electronic properties of the electrode and the channel region separately during deriving the lateral SBH. This coupling makes the Ohmic contact difficult to occur because the Fermi level is pinned below the CBM of MoS_2_. Therefore, caution must be taken for any lateral Ohmic contact predicted by the energy band analysis, and a further quantum transport calculation is necessary to obtain a more reliable interface. Actually, the Ohmic contact in ML phosphorene-Pd contact derived from the energy band analysis also turns out to be artificial in terms of the quantum transport simulations^61^.

If the SBH appears in the vertical direction, the coupling between metal and MoS_2_ has been taken into account in the energy band calculations because the metal and semiconductor parts are treated a whole. In this case, it appears that the quantum transport simulation will give similar SBH. We calculate the transport properties of ML and BL MoS_2_ with Pt electrodes. As the lattice mismatch between MoS_2_ and Pt supercell is small (~1.2%), we only consider Model I in which the lattice constant of ML/BL MoS_2_ is adapted to that of Pt supercell. As shown in Fig. S1, transport gaps of 1.34 and 1.03 eV are observed for ML and BL MoS_2_-Pt interfaces in the transmission spectra. The Fermi level in BL MoS_2_-Pt contact is apparently closer to the VBM, having a hole SBH of 0.32 eV, which is in indeed good agreement with the one (0.345 eV) from the energy band analysis. It appears that the coupling between Pt and BL MoS_2_ has been taken into account in the energy band calculations. It is notable that the energy band analysis and the quantum transport simulations also give similar *p*-type SBHs for ML WSe_2_-Pt interface (0.28 and 0.34 eV, respectively)^62^. However, the Fermi level of ML MoS_2_-Pt contact is located nearly in the middle of the transport gap (slightly closer to the CBM of ML MoS_2_), showing a midgap SBH. This is not in consistent with the energy band analysis, which favors a *p* doping of ML MoS_2_ with a hole SBH of 0.520 eV. The story becomes more complicated as the experimental observations show electron SBH of ~0.23 eV for 3–8 layers MoS_2_-Pt interface^21^. The origin of the controversy among the energy band analysis, quantum transport simulations, and experiments remains unclear and more studies on the MoS_2_-Pt system are desirable. It is interesting to mention that, in the MoS_2_-Pd system, both *n*- and *p*-doped characteristics of MoS_2_ have been reported^63^,^64^,^65^. It is well known that Pt has a larger work function than Pd (6.1 eV vs 5.6 eV)^51^, and generally Pt can induce *p* doping more easily. It appears that the possibility of *p* doping of ML and BL MoS_2_ by Pt contact cannot be excluded completely.

## Discussions

**Figure 10:**
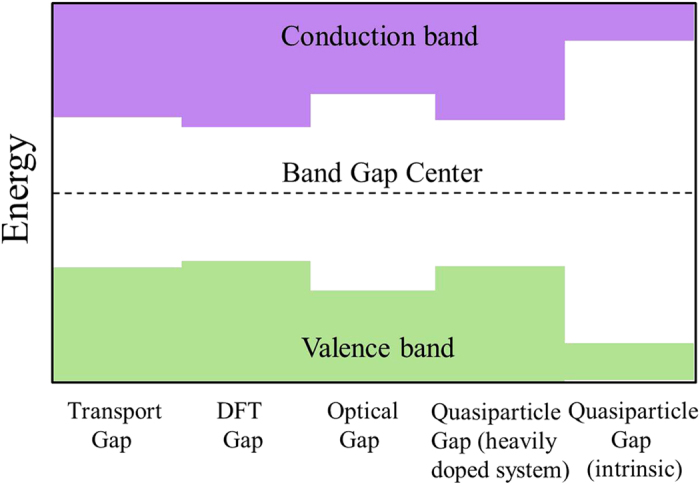
A schematic diagram for the size relation of the five common band gaps of a 2D semiconductor.

There are four types of commonly used band gap for a 2D semiconductor: transport gap, quasiparticle gap (dominated by electron-electron correlation), optical gap (dominated by strong exciton effects), and DFT gap (single electron approximation). Taking ML/BL phosphorene as an example, the four band gaps are: 0.98/0.71^57^, 2.0/1.3^58^, 1.30/0.70 , and 0.91/0.60 eV^59^. Apparently, the DFT band gap and optical gap are the closest to the transport gap because the 2D channel semiconductor is doped by carrier. In addition to doping by electrode, the 2D semiconductor channel is also subject to electrostatic doping by gate. This is another cause why many-electron effects are strongly depressed of the 2D channel semiconductor. However, the transport gaps are still about 10% slightly larger than their respective DFT gaps in phosphorene^66^, suggestive of the existence of weak many-electron effects with about 10% correction in doped phosphorene, which is one order of magnitude smaller than that in intrinsic phosphorene. Actually, the band gap of a heavily doped silicene is 0.34 and 0.38 eV at the DFT and *GW* level, respectively, consistent with a correction of about 10% upon the inclusion of the many-body effects^38^. From a physical point of view, the transport gap of a 2D semiconductor should equal to the quasipartical band gap of heavily doped system, which is slightly larger than the DFT band gap. Figure 10 illustrates the size relation of these common band gaps. Hence, a small correction (increase by about 10%) to the DFT CBM and VBM appears to be required to obtain the accurate CBM and VBM positions of a doped 2D semiconductor and thus get the accurate SBH at the interface.

## Conclusion

In summary, we provide the first comparative study of the interfacial properties of monolayer and bilayer MoS_2_ on Sc, Ti, Ag, Pt, Ni, and Au surfaces by using different theoretical approaches. A comparison between the calculated and observed Schottky barrier heights suggests that many-electron effects are strongly depressed but do not vanish and the transport gap of a device depends on the DFT-GGA band gap (a minor correction is still needed) rather than the quasiparticle band gap. Such a depression of many-electron effects can be applied to a general metal-2D semiconductor interface. In generally, the Schottky barrier heights are decreased from ML MoS_2_-metal interfaces to BL MoS_2_-metal interfaces due to the interlayer coupling, implying that BL MoS_2_ with a higher electron injection efficiency is probably more suitable for a transistor than ML MoS_2_ given the same gate controllability. Most strikingly, we find that DFT energy band calculations are unable to reproduce the experimental Schottky barrier heights in some cases and give incorrect Ohmic contact prediction because the Fermi level pinning has not been fully taken into account. In the interface study between other 2D material and metal, such a shortcoming remains. To solve such a problem, a higher level *ab initio* quantum transport calculation based on a two-probe model is desired.

## Additional Information

**How to cite this article**: Zhong, H. *et al.* Interfacial Properties of Monolayer and Bilayer MoS_2_ Contacts with Metals: Beyond the Energy Band Calculations. *Sci. Rep.* **6**, 21786; doi: 10.1038/srep21786 (2016).

## Supplementary Material

Supplementary Information

## Figures and Tables

**Table 1:** Calculated interfacial properties of ML and BL MoS_2_ on metal surfaces.

Metal	Mismatch	*W*_M_(eV)	ML MoS_2_						BL MoS_2_					
			*d*_*S*-M_(Å)	*E*_b_(eV)	*W*(eV)	Δ*E*_*f*_(eV)	*Φ*_*v*_(eV)	*Φ*_*L*_(eV)	*d*_*S*-M_(Å)	*E*_b_(eV)	*W*(eV)	Δ*E*_*f*_(eV)	*Φ*_*v*_(eV)	*Φ*_*L*_(eV)
Sc3.308(Å)	4.485%	3.593	1.786	1.181	4.369	−0.881	0.000	0.000	1.783	1.182	4.306	−0.944	0.000	0.000
							0.000	(0.539)^*GW*^					0.000	(0.000)^*GW*^
	1.290%				4.19			0.000			4.300	−0.950		0.000
								(0.362)^*GW*^						(0.000)^*GW*^
Ti2.951(Å)	6.791%	4.427	1.557	1.812	4.597	−0.653	0.000	0.187	1.560	1.848	4.616	−0.634	0.000	0.096
								(0.3–0.35)^b^					—	(0.065) d
							(0.000)^a^	(0.33)^c^					—	(0.276)^*GW*^
							—	(0.731)^*GW*^					0.000	–
	2.990%				4.626		0.000	0.216			4.681			0.161
								(0.796)^*GW*^						(0.341)^*GW*^
Ag5.778(Å)	5.367%	4.489	2.961	0.503	4.662	−0.588	0.212	0.000	2.917	0.547	4.763	−0.487	0.138	0.000
Ni4.984(Å)	9.112%	5.222	2.094	0.830	5.001	−0.249	0.633	0.000	2.097	0.729	5.102	−0.148	0.612	0.000
Au5.768(Å)	5.185%	5.226	3.405	0.307	5.173	−0.077	0.763	0.000	3.325	0.354	5.187	−0.063	0.667	0.000
							(0.88)^e^	(0.000)^e^					—	—
Pt5.549(Å)	1.191%	5.755	2.476	0.570	5.444	0.194	0.520	0.000	2.438	0.634	5.476	0.226	0.345	0.000
							(0.770)^f^							

The experimental cell parameters of the surface unit cells shown in Fig. 1 for various metals are given under the metals. The corresponding lattice mismatches are given. The equilibrium distance d_S-M_ is the averaged distance between the surface S atoms of MoS_2_ and the relaxed positions of the topmost metal layer in the z direction. E_b_ is the binding energy per surface S atom between MoS_2_ and a given surface. W_M_ and W are the calculated work functions for clean metal surface and metal surface adsorbed by MoS_2_, respectively. Φv and Φ_L_ are the vertical and lateral SBH at the DFT level, respectively, of a MoS_2_ transistor (see Fig. 7(c)); the SBH obtained in other DFT calculations, the GW-corrected SBHs, and the measured SBH are given below them in parenthesis for comparison. ΔE_f_ is the Fermi level shift of 2D MoS_2_. The corresponding values for Sc and Ti surfaces in small mismatch are also given. Caution must be taken for the data of ML and BL MoS_2_-Ni contacts due to the large lattice mistmach (9.1%) limited by the computational resource.

^a^DFT values from refs. 22,39.

^b^Experimental value^23^.

^c^DFT value from ref. 23,39.

^d^Experimental value at a low temperature in ref. 26.

^e^DFT value from ref. 28.

^f^The SBH for ML MoS_2_-Pt is hole SBH and the DFT value from ref. 28.

**Table 2:** Tunneling barrier height Δ*V*, width *w*_B_, and probabilities (*T*_B_) through the ML (BL) MoS_2_-metal interfaces.

Metal	ML MoS_2_			BL MoS_2_		
	Δ*V*	*w*_B_	*T*_B_	Δ*V*	*w*_B_	*T*_B_
	(eV)	(Å)	(%)	(eV)	(Å)	(%)
Sc	0.000	0.000	100	0.000	0.000	100
Ti	0.000	0.000	100	0.000	0.000	100
Ag	3.003	0.916	19.68	2.911	0.904	20.61
Ni	0.785	0.327	74.33	0.822	0.336	73.20
Au	4.697	1.374	4.74	4.585	1.356	5.11
Pt	1.810	0.458	53.21	1.871	0.517	48.47
