# Supplementary material for: Interfacial Properties of Monolayer and Bilayer MoS2 Contacts with Metals: Beyond the Energy Band Calculations
Source: Sci Rep. 2016 Mar 1;6:21786. doi: 10.1038/srep21786 (PMC4772071; doi:10.1038/srep21786)
Supplement: Supplementary Information [file srep21786-s1.pdf]

Interfacial Properties of Monolayer and Bilayer MoS_2 Contacts with Metals: Beyond the Energy Band Calculations

Hongxia Zhong,[1,†] Ruge Quhe,[1,3,†] Yangyang Wang,[1,5] Zeyuan Ni,[1] Meng Ye,[1] Zhigang Song,[1] Yuanyuan Pan,[1] Jinbo Yang,[1,2] Li Yang,[4] Lei Ming,[3] Junjie Shi,[1,*] and Jing Lu[1,2,*]

[1]State Key Laboratory for Mesoscopic Physics and Department of Physics, Peking University, Beijing 100871, P. R. China

[2]Collaborative Innovation Center of Quantum Matter, Beijing 100871, P. R. China

[3]State Key Laboratory of Information Photonics and Optical Communications, Beijing University of Posts and Telecommunications & School of Science, Beijing 100876, China

[4]Department of Physics, Washington University in St. Louis, St. Louis, Missouri 63130, USA

[5]Department of Nuclear Science and Engineering and Department of Materials Science and Engineering, Massachusetts Institute of Technology, Cambridge, Massachusetts 02139 , USA

[†]These authors contributed equally to this work.

Email: jjshi@pku.edu.cn; jinglu@pku.edu.cn

Table S1. The work functions *W* for metal surface adsorbed by MoS_2. The vertical Φ_V and lateral Φ_L SBH at the DFT level of a MoS_2 transistor (see Fig. 7(c)) when the lattice constants of metal surfaces are adjusted to that of MoS_2.

Metal	ML MoS_2			BL MoS_2		
	W (eV)	Φ_V (eV)	Φ_L (eV)	*W* (eV)	Φ_V (eV)	Φ_L (eV)
Sc	4.161	0.000	0.000	4.310	0.000	0.000
Ti	4.561	0.000	0.151	4.411	0.000	0.000
Ag	4.625	0.215	0.000	4.558	0.138	0.000
Ni	5.115	0.705	0.000	5.240	0.820	0.000
Au	5.058	0.813	0.000	5.211	0.559	0.000
Pt	5.432	0.648	0.000	5.079	0.659	0.000

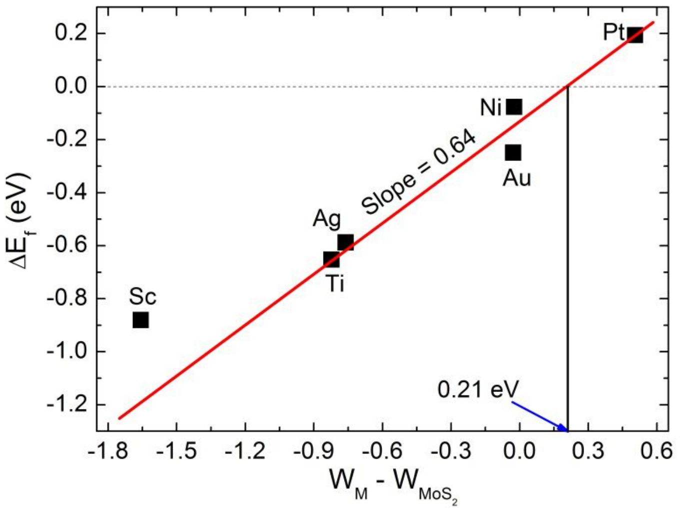

FIG. S1. Calculated Fermi level shift ΔE_f as a function of $W_{\mathrm{M}} - W_{\mathrm{MoS_2}}$, the difference between the clean metal and ML MoS_2 work functions at the DFT level. $W_{\mathrm{M}} - W_{\mathrm{MoS_2}} = 0.21$ eV is the cross point from *n*- to *p*-type doping. The red line is the fitting curve to the calculated points, and the slope of the line is 0.64.

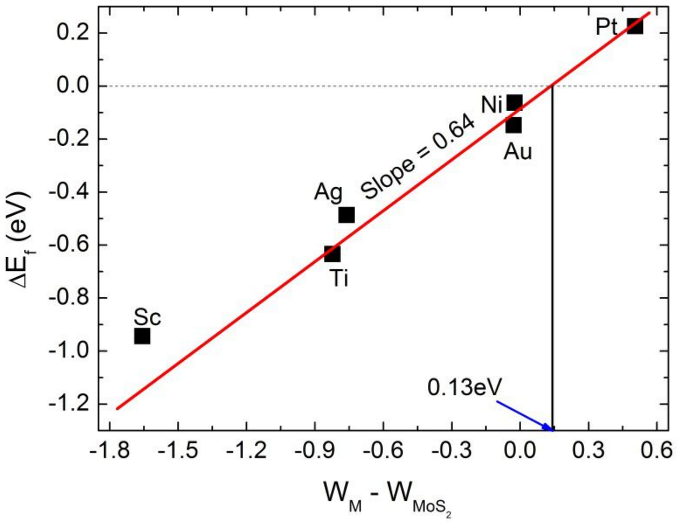

FIG. S2. Calculated Fermi level shift ΔE_f as a function of $W_{\mathrm{M}} - W_{\mathrm{MoS_2}}$, the difference between the clean metal and BL MoS_2 work functions at the DFT level. $W_{\mathrm{M}} - W_{\mathrm{MoS_2}} = 0.13$ eV is the cross point from *n*- to *p*-type doping. The red line is the fitting curve to the calculated points, and the slope of the line is 0.64.
